# Further Evidence on Trace Element Imbalances in Haemodialysis Patients—Paired Analysis of Blood and Serum Samples

**DOI:** 10.3390/nu15081912

**Published:** 2023-04-15

**Authors:** Rui Azevedo, Davide Gennaro, Mary Duro, Edgar Pinto, Agostinho Almeida

**Affiliations:** 1 LAQV/REQUIMTE, Department of Chemical Sciences, Faculty of Pharmacy, University of Porto, 4050-313 Porto, Portugal; 2 Department of Pharmaceutical and Pharmacological Sciences, University of Padua, 35131 Padova, Italy; 3 FP-ENAS—Fernando Pessoa Energy, Environment and Health Research Unit, Fernando Pessoa University, 4249-004 Porto, Portugal; 4 Laboratório de Análises Clínicas Dra. Matilde Sampaio, 5200-216 Mogadouro, Portugal; 5 Laboratório de Análises Clínicas Vale do Sousa, 4560-547 Penafiel, Portugal; 6 Department of Environmental Health, ESS, Polytechnic of Porto, 4200-072 Porto, Portugal

**Keywords:** haemodialysis, kidney failure, trace elements, ICP-MS

## Abstract

Previous studies have shown that haemodialysis patients have an increased risk of trace element imbalances. Most studies have determined the concentration of trace elements in serum only, but most trace elements are not uniformly distributed between plasma and blood cells, which justifies separate analysis of the different compartments. In this study, we determined both the serum and whole blood concentration of a wide panel of trace elements (Li, B, Mn, Co, Ni, Cu, Zn, Se, Rb, Sr, Mo, Cd, Pb) in haemodialysis patients and compared them with those of a control group. Whole blood and serum samples were collected during routine laboratory testing of patients undergoing chronic haemodialysis. For comparison purposes, samples from individuals with normal renal function were also analysed. Statistically significant differences (*p* < 0.05) were found between the two groups for whole blood concentrations of all analysed elements except Zn (*p* = 0.347). For serum, the difference between groups was statistically significant for all elements (*p* < 0.05). This study confirms that patients on haemodialysis tend to present significant trace element imbalances. By determining the concentration of trace elements in both whole blood and serum, it was shown that chronic haemodialysis may affect intra- and extracellular blood compartments differently.

## 1. Introduction

Chronic kidney disease (CKD) is defined as abnormalities of kidney structure or function present for more than 3 months, with implications for the individual’s health [1]. CKD affects more than 10% of the general population worldwide, which corresponds to more than 800 million individuals [2]. It is more prevalent in older, female individuals and is usually accompanied by several comorbidities, such as diabetes, hypertension and cardiovascular diseases [2,3]. 

The majority of CKD cases are irreversible and have a lifelong progression. Thus, treatment is directed at slowing the progression to kidney failure [1]. Kidney failure is characterised by a glomerular filtration rate (GFR) < 15 mL/min/1.73 m^2^ and requires renal replacement therapy (RRT), most often haemodialysis (HD), peritoneal dialysis or kidney transplantation [1]. In 2010, 2.6 million individuals received RRT worldwide, and it is estimated that at least 4.9 million individuals will need RRT by 2030 [4].

Haemodialysis is the most common modality of RRT [5,6]. However, while HD offers years of life, it is a chronic therapy that affects the lifestyle, eating habits and overall quality of life of patients [7,8,9] and their families and caregivers [10,11,12]. 

In HD, a machine pumps blood through a tube system from the patient’s vein to the dialyser (“artificial kidney”), where the blood is cleared through an artificial semipermeable membrane that separates it from the dialysate, then returns it to the patient’s body [13,14]. The semipermeable membrane is the key component of the entire system, regardless of which of the two the basic processes of solute and fluid movement are used: (i) diffusive clearance, where solutes move down their concentration gradient from areas of high concentration to low concentration, and (ii) convective clearance (hemofiltration or ultrafiltration), where a higher hydrostatic pressure in the blood compartment than in the dialysate compartment is used to promote the passage of blood fluid into the dialysate, with the solutes moving along with the water, a modality that requires the concomitant administration of a replacement fluid to compensate for the large fluid removal during hemofiltration [13,14].

Previous studies have shown that HD patients are at an increased risk of trace element imbalances [15,16,17,18,19,20,21,22,23]. While the concentration of essential trace elements may be decreased due to the inflammatory nature of the disease [24,25,26], lower food intake due to uremic anorexia or dietary restrictions [27,28,29] or losses during dialysis sessions [30], the concentration of non-essential/toxic trace elements may be increased due to loss of ability to excrete them in urine [31], among other factors.

Most studies on trace element imbalances in HD patients have analysed their serum/plasma concentration [15,16,17,18,19,20], with only a few focusing on whole blood analysis [22,23]. However, in most cases, circulating trace elements are not uniformly distributed between plasma and cellular blood components. For example, only about 4% of circulating Mn is present in plasma, with 66% found in erythrocytes and the remaining 30% in leucocytes and platelets [32]. Another example is Pb, where approximately 99% of circulating Pb is found in erythrocytes [33]. This means that changes observed in the serum of HD patients may not be present in whole blood or its cellular components, and vice versa.

Previously, we observed lower serum concentrations of Mn, Cu, Zn, Se, Rb and Ba and higher concentrations of Al, Co, Ni, Sr, Mo, Cd and Pb in a group of HD patients compared to a control group (individuals without renal impairment) [34]. The present study aimed to further investigate this issue by measuring both serum and whole blood concentrations of several essential and non-essential/toxic trace elements in a cohort of HD patients and comparing them to a control group.

## 2. Materials and Methods

### 2.1. Study Design and Sample Collection

This work is a comparative study of trace element concentrations in serum and whole blood of HD patients and a control group of individuals without renal impairment. Whole blood (*n* = 108) and serum (*n* = 88) samples were collected during routine laboratory testing from individuals undergoing regular HD therapy at a Dialysis Centre in northern Portugal. The control group was established by using whole blood (*n* = 59) and serum (*n* = 20) samples from apparently healthy individuals who attended the same Clinical Laboratory and met the standard laboratory analytical criteria for normal kidney function.

Whole blood samples were collected by conventional venepuncture into BD (Franklin Lakes, NJ, USA) Vacutainer™ polyethylene terephthalate (PET) tubes (K_2_EDTA as anticoagulant) or Greiner Bio-One (Madrid, Spain) VACUETTE^®^ PET tubes (sodium heparin as anticoagulant), special for trace element analysis, and kept refrigerated until analysis. Serum samples were obtained by collecting blood through conventional venepuncture into VACUETTE^®^ Z Trace Elements no-additive PET tubes. After allowing the specimen to clot for 30 min, the serum was separated into previously decontaminated Eppendorf tubes and kept refrigerated until analysis.

Data anonymisation was ensured by removing all patient information except sex and age. The study was approved by the Ethics Committee of the Faculty of Pharmacy of the University of Porto (Parecer Nº 38-06-2019).

### 2.2. Reagents 

All solutions were prepared with ultrapure water (>18.2 MΩ·cm at 25 °C) obtained with a Sartorius Arium^®^ pro water purification system (Gottingen, Germany). Nitric acid (HNO_3_, 67–69% *w*/*w* TraceMetal^®^ Grade) was obtained from Fisher Scientific (Leicestershire, UK). Triton X-100, butanol (>99.0%) and multi-element stock solutions (10 mg/L) Periodic Table Mix 1, 2 and 3 were obtained from Sigma-Aldrich (St. Louis, MO, USA). Copper and Zn single-element stock solutions (1000 mg/L) were obtained from SCP Science (Quebec, QC, Canada). All laboratory ware (bottles, tubes, volumetric flasks) was made of polypropylene or high-density polyethylene (HDPE) and was properly decontaminated by immersion in a 10% *v/v* HNO_3_ solution for at least 24 h, followed by abundant rinsing with ultrapure water and drying at room temperature under dust-free conditions.

### 2.3. Laboratory Procedures

Whole blood and serum samples were analysed at the Laboratory of Applied Chemistry (Trace Element Analysis Unit) of the Faculty of Pharmacy, University of Porto, Portugal, using properly validated inductively coupled plasma mass spectrometry (ICP-MS) analytical procedures. The instrument was an iCAP™ Q (Thermo Fisher Scientific, Bremen, Germany), equipped with a Meinhard^®^ (Golden, CO) TQ+ quartz concentric nebuliser, a Peltier cooled, high purity quartz, baffled cyclonic spray chamber and a demountable quartz torch with a 2.5 mm i.d. quartz injector. The interface consisted of two Ni cones (sampler and skimmer). High-purity argon (99.9997%) supplied by Gasin (Matosinhos, Portugal) was used both as nebuliser gas and plasma gas. Prior to each analytical run, the instrument was tuned for maximum sensitivity and signal stability and for minimal formation of oxides and double charged ions. The main operating parameters of the ICP-MS instrument were nebuliser gas flow, 1.16 L/min; auxiliary gas flow, 0.79 L/min; plasma gas flow, 13.9 L/min; radio frequency generator power, 1550 W; dwell time, 10 ms.

For serum analysis, a procedure based on Goullé et al. [35] was used. Briefly, samples were diluted 1:10 with a diluent solution containing 0.65% *v/v* HNO_3_, 0.01% *v/v* Triton X-100, 0.5% *v/v* butanol and internal standards (IS) at 10 µg/L (added by proper dilution of Periodic Table Mix 3 for ICP). For whole blood analysis, a similar procedure based on Goullé et al. [35] was used. Briefly, samples were diluted 1:15 with a diluent solution containing 0.65% *v/v* HNO_3_, 0.1% *v/v* Triton X-100, 0.5% *v/v* butanol and IS at 10 µg/L (added by proper dilution of Periodic Table Mix 3 for ICP).

For Li, B, Mn, Co, Ni, Se, Rb, Sr, Cd and Pb, an 8-point calibration curve (1, 5, 10, 25, 50, 100, 250 and 500 µg/L) was generated with standard solutions prepared by appropriate dilution of Periodic Table Mix 1 in 2% HNO_3_. For Mo, an 8-point calibration curve (0.05, 0.25, 0.5, 1.25, 2.5, 5, 12.5 and 25 µg/L) was generated with standard solutions prepared by appropriate dilution of Periodic Table Mix 2 in 2% HNO_3_. For Cu and Zn, a 5-point calibration curve (0.5, 1.0, 1.5, 2.0 and 5.0 mg/L) was generated with mixed standard solutions prepared by appropriate dilution of single-element standard stock solutions in 2% HNO_3_. Calibration solutions were then diluted 1:10 (for serum analysis) or 1:15 (for whole blood analysis) with the diluent solution, as the samples. The elemental isotopes ^7^Li, ^11^B, ^55^Mn, ^59^Co, ^60^Ni, ^65^Cu, ^66^Zn, ^82^Se, ^85^Rb, ^88^Sr, ^98^Mo, ^111^Cd, ^206^Pb, ^207^Pb and ^208^Pb were measured for analytical determinations and ^89^Y, ^141^Pr and ^159^Tb were monitored as IS.

After complete mixing on a vortex mixer, the diluted samples and calibration standards were presented to the ICP-MS instrument using a CETAC ASX-520 auto sampler (Teledyne CETAC Technologies, Omaha, NE). Samples were analysed in random order to avoid sequence effects. For analytical quality assurance, repeated analysis (at the beginning, middle and end of each analytical run) of Seronorm™ Trace Elements Serum L-1 and L-2 and Seronorm™ Trace Elements Whole Blood L-1, L-2 and L-3 (obtained from SERO AS, Billingstad, Norway) was performed. The results obtained are presented in Supplementary Materials (Table S1).

### 2.4. Statistical Analysis

Statistical analysis was performed using SPSS Statistics v.27.0 (IBM Corporation, Armonk, NY, USA). Data were summarised as mean, standard deviation (SD) and range. For statistical calculations, results below the limit of detection (LD; Table S2) were imputed as $LD/\sqrt{2}$. The normality of data distribution was assessed using Shapiro–Wilk and Kolmogorov–Smirnov tests. Variables non-normally distributed were Ln transformed and data normality was reassessed. Homogeneity of variance was checked using Levene’s test. For variables with normal distribution, Student’s *t*-test or Welch’s *t*-test was used to determine the significance of differences in trace elements concentration between HD patients and the control group. For the variables which remained non-normally distributed after Ln transformation (whole blood Zn and serum Cd), the Mann–Whitney U test was used. Hedges’ g was used to estimate the effect size of chronic HD on trace element concentration. Statistical significance was set at *p* < 0.05.

## 3. Results

The mean (SD) age of participants in the whole blood analysis study was 72 (12) years for the HD patient group vs. 64 (19) years for the respective control group; in the serum analysis study it was 69 (14) years for the HD patient group vs. 64 (10) years for the respective control group. The male/female ratio was 61/39 in the HD patient group for whole blood analysis vs. 34/66 in the control group, and 57/43 in the HD patient group for serum analysis vs. 50/50 in the control group.

Statistically significant differences were found between HD patients and controls for all elements (*p* < 0.05), except for Zn (*p* = 0.347). Specifically, Li, B, Mn, Ni, Sr, Mo, Cd and Pb concentrations were significantly higher in HD patients (*p* < 0.05), while Co, Cu, Zn, Se and Rb were significantly lower (*p* < 0.001; Table 1).

In serum, statistically significant differences were found between HD patients and controls for all elements analysed (*p* < 0.05). Specifically, Li, B, Co, Ni, Sr, Mo, Cd and Pb concentrations were significantly higher in HD patients (*p* < 0.05), while Mn, Cu, Zn, Se and Rb concentrations were significantly lower (*p* < 0.001). The Cu/Zn ratio was also significantly higher in HD patients (*p* = 0.004; Table 2). 

## 4. Discussion

The results of our study showed a lower concentration of Mn in serum samples from patients in HD compared to a control group of individuals without renal impairment (*p* < 0.001). In their systematic review with meta-analysis, Tonelli et al. (2009) reported lower Mn concentration in HD patients [21]. More recently, Almeida et al. (2020) also found a significantly lower serum Mn concentration in HD patients, but Stojsavljević et al. (2022) reported increased Mn concentrations [15,34]. It should be noted, as mentioned before, that 96% of circulating Mn is found in erythrocytes, leucocytes and platelets, while only 4% is present in plasma [32]. The small fraction of circulating Mn can be partially lost during the HD process or increased due to contamination of the dialysis fluid [30], which may explain the different results reported in the literature. Furthermore, in our study the mean concentration of Mn in whole blood was significantly higher in HD patients than in the control group (*p* = 0.025). Two previously published studies also reported the same finding [23,36], while two others found no statistically significant differences [37,38]. Interestingly, one of these latter studies found Mn depositions in the basal ganglia of HD patients, but not in patients who underwent kidney transplantation, patients on peritoneal dialysis or healthy controls [37]. Additionally, despite not having measured Mn, Ninić et al. (2018) found a significantly increased (*p* = 0.011) gene expression of mitochondrial superoxide dismutase (SOD, a Mn-dependent enzyme) in peripheral blood mononuclear cells of HD patients [39].

Despite the great heterogeneity of results in the literature regarding Cu, Tonelli et al. (2009) reported a higher Cu concentration in HD patients in their meta-analysis [21]. In the present study, we found a significantly decreased concentration of Cu in both whole blood and serum of HD patients compared to the control group (*p* < 0.001). In a previous study by our group, Almeida et al. (2020) also found a lower serum Cu concentration in a group of HD patients from northern Portugal [34]. Other studies have shown mixed results; Some found no differences in serum [16,18,38] or whole blood Cu concentration [23,36], while others found an increased [15,40,41] or a decreased serum concentration [42]. Copper is mainly excreted in bile [43], which means that loss of renal function is unlikely to significantly interfere with Cu excretion, at least directly. Losses during the HD session [30] and/or low food intake [27] may explain the decreased Cu concentration in chronic HD patients found in some studies. However, oxidative stress and chronic inflammation are two hallmarks of kidney failure and HD [25,26], and Cu is a trace element strongly involved in protection against oxidative stress [43], which may explain the increased concentration of Cu in HD patients found in several studies. Specifically, Cu is a cofactor of the cytoplasmic and extracellular isoforms of SOD, an important enzyme for redox homeostasis [44], whose activity and gene expression have been shown to be increased in erythrocytes [41] and peripheral blood mononuclear cells [39] of HD patients, respectively.

Tonelli et al. (2009) reported significantly decreased Zn concentrations in HD patients in their meta-analysis [21]. This seems to be a consistent finding with regard to serum Zn concentration [15,38,41,42,45], which we also observed both in the present study (*p* < 0.001), and in a similar study by our group previously published [34]. With regard to the concentration of Zn in whole blood, there are few published studies. For this specimen, we found no statistically significant difference between HD patients and controls (*p* = 0.347), despite a slightly lower mean Zn concentration in HD patients. Oruc et al. (2022) reported a median concentration (interquartile range) of 3.48 (3.28–3.99) and 3.71 (3.37–3.98) mg/L in HD patients and controls, respectively, with no statistically significant difference (*p* = 0.240) between groups [36]. On the other hand, Prodanchuk et al. (2013) found a significantly increased mean (SD) concentration of Zn: 5.81 (0.14) mg/L in HD patients vs. 4.93 (0.07) mg/L in the control group (*p* < 0.001) [23]. However, it is important to note that the Zn concentration in whole blood found in the first study was much lower than in the second and in ours. Zinc balance studies during RRT have provided variable results [30]. Only ~0.1% of total body Zn stores are present in plasma, either weakly bound to albumin (~80%) or strongly bound to α2-macroglobulin (~20%) [46]. Chronic systemic inflammation, which is commonly observed in HD patients [25], may affect serum Zn [24]. In addition to lower dietary intake [27] and potential interactions with Fe supplements [47], all these factors may collectively contribute to the lower serum Zn concentration commonly seen in HD patients. 

The serum Cu/Zn ratio was significantly higher (*p* = 0.004) in HD patients compared to the control group. Inflammation and oxidative stress, two hallmark features of renal failure and chronic HD [25,26], have been shown to influence the serum concentration of both elements, markedly increasing Cu concentration and decreasing Zn concentration [48]. A higher serum Cu/Zn ratio has been associated with an increased risk of all-cause mortality [48], heart failure [49], infection [50], colorectal cancer [51], impaired glomerular filtration rate [52], decreased handgrip strength [53] and impaired glycaemic control [52]. In HD patients, an increased serum Cu/Zn ratio has been shown to be associated with higher levels of total and LDL cholesterol [54] and carotid artery atherosclerosis [40], thus an increased risk of cardiovascular disease.

For Se, we found a significantly lower concentration in both whole blood and serum of HD patients (*p* < 0.001), as reported by Tonelli et al. (2009) in their meta-analysis [21] and in a previous study by our group [34]. Other recent studies have reported similar results for Se in serum [15,38,41,45]. In whole blood, one study found a decreased concentration of Se in HD patients compared to healthy controls [36], while another study found no statistically significant differences (*p* = 0.217) [22]. Selenium is another trace element strongly involved in the regulation of the inflammatory process and redox status [55], and its serum concentration seems to be inversely related to the inflammatory status [24]. As mentioned above, increased oxidative stress is commonly observed in patients with renal failure on chronic HD [26]. Several glutathione peroxidase (GPx) isoforms, essential for protection against oxidative damage, are Se-dependent [55]. Some previously published studies observed a significantly decreased GPx activity in HD patients [41,56], but others did not [57]. The causes of this Se deficiency will be several, including dietary restrictions [58]. Additionally, Selenium appears to be lost in the dialysis fluid during the dialysis process [30]. It is important to mention that Se was the only essential trace element that was shown to be strongly and independently associated with an increased risk of death and hospitalisation in a prospective cohort study by Tonelli et al. (2018) [20]. 

Cobalt’s only known biological function is as a component of vitamin B12 (cobalamin), while other Co compounds are toxic to humans [59]. In this study, we found a significantly decreased whole blood Co concentration (*p* < 0.001) and a significantly increased serum Co concentration in HD patients (*p* < 0.001). Only one small study showing no statistically significant differences in Co concentration between HD patients and a control group was included in the review by Tonelli et al. (2009) [21]. In a previous study by our group, an increased serum Co concentration in HD patients was also found [34], while Stojsavlkević et al. (2022) reported a decreased concentration [15]. In whole blood, two previously published studies found a significantly higher (*p* < 0.001) Co concentration in HD patients [22,36] while in another study the difference was not statistically significant (*p* = 0.501) [23]. Vitamin B12 and its biologically inactive analogues are found in circulation bound to transcobalamin or haptocorrin [60]. Cellular uptake of vitamin B12 is achieved through the interaction of a cell surface receptor with transcobalamin bound to vitamin B12 (holotranscobalamin) [60]. Holotranscobalamin is filtered by the kidney and is often increased in patients with renal failure [61]. Vitamin B12 does not seem to be lost during HD [30,62], which means that changes in serum Co concentration will result from direct Co losses, contamination of the dialysis fluid due to its contact with Co-containing metallic alloys, or variations in circulating levels of vitamin B12. Despite the increased levels of holotranscobalamin, some studies have shown a lower cellular uptake of vitamin B12 in patients with CKD [63,64], which could explain the lower levels of Co found in whole blood of HD patients.

There are very few studies on Mo status in HD patients. In this study, a significantly increased (*p* < 0.001) Mo concentration was observed in both whole blood and serum of HD patients. In the meta-analysis by Tonelli et al. (2009), only one study with Mo results was included, which reported increased Mo concentrations in HD patients [21] and Almeida et al. (2020) also found higher serum Mo concentrations in HD patients [34]. Molybdenum is mainly excreted in urine [65], which may explain the increased Mo concentrations observed in HD patients. On the other hand, the dialysis process appears to be able to remove Mo from circulation [66]. 

Cadmium is a well-established human carcinogen [67]. In this study, we observed a significantly increased Cd concentration in both whole blood and serum of HD patients (*p* < 0.001). Consistent with our findings, Tonelli et al. (2009) also reported increased Cd levels in HD patients in their meta-analysis [21], as did Almeida et al. (2020) in the serum of a cohort of HD patients from northern Portugal [34]. With only one exception [15], all other recent studies have also reported increased Cd concentrations in both whole blood [22,23,36,68,69,70] and serum [71] of HD patients. In circulation, Cd is mostly found in erythrocytes bound to metallothioneins and other SH-rich proteins and peptides [72,73]. Due to their low molecular weight, serum metallothioneins are filtered and extensively reabsorbed in the kidneys, causing Cd accumulation [74]. As a result, the kidneys constitute the main body reservoir of Cd, with a half-life of several years [75,76]. Loss of renal function impairs the main route of Cd elimination from the circulation [77], which may explain the increased Cd concentrations found in HD patients.

Lead is a toxic trace element with deleterious effects on virtually all functions of the human body [78]. In the present study, a significantly increased concentration of Pb was found in both whole blood and serum of HD patients (*p* < 0.001). This was also reported by Tonelli et al. (2009) in their meta-analysis [21], and has been confirmed in several other more recent studies, which reported increased Pb levels in both whole blood [22,23,36,38,70,79] and serum [15,34,71] of HD patients. In adult humans, approximately 90% of the total body burden of Pb is found in bone [80]. Mineral bone disorders are often seen in patients with renal failure, mainly due to their advanced age, abnormal metabolism of calcium, phosphate, vitamin D and parathyroid hormone, soft tissue calcification and abnormal bone remodelling and mineralisation [81,82]. In postmenopausal women, bone mineral density progressively decreases while circulating Pb levels increase [83]. It is possible that the increased Pb levels found in HD patients are due to Pb mobilisation during bone resorption caused by an altered bone metabolism. 

Serum and whole blood concentrations of Li, B, Ni and Sr were all significantly increased in HD patients (*p* < 0.05) compared to controls. In the meta-analysis by Tonelli et al. (2009) mixed results were reported for B and Ni, and there is no reference to studies on Li or Sr [21]. In the previous study by our group, an increased serum concentration of Ni and Sr in HD patients was also reported [34]. Other recent studies have reported an increased Li and B concentration in whole blood [23,36] and an increased Ni and Sr concentration in both whole blood [23,36] and serum [15,84], as observed in the present study. All of these trace elements are excreted mainly in the urine [85,86,87,88]. Therefore, the loss of renal function may explain the increased concentrations of Li, B, Ni and Sr observed in HD patients. Additionally, most of the body content of Sr is present in the bone [89]. Mineral bone disturbances, commonly seen in patients with renal failure, as mentioned above, may cause mobilisation of bone Sr (as described for Pb) and explain the increased levels of circulating Sr observed in HD patients. The same might be true for B because it also accumulates in bone [90]. 

Lastly, we found a significantly decreased concentration of Rb in both whole blood and serum of HD patients (*p* < 0.001). Data on changes in Rb concentrations in HD patients are very scarce. Almeida et al. (2020) and Stojsavlkević et al. (2022) both reported decreased serum concentrations in HD patients compared to a healthy control group [15,34]. Rubidium appears to be lost in the dialysis fluid during the dialysis process [66,91] and patients with renal failure also appear to have a decreased intake of Rb [92]. Together, these factors may explain the decreased Rb concentrations in HD patients.

Overall, the results of this study show that chronic HD appears to affect the relative distribution of various trace elements between plasma and the intracellular compartment of blood cells. While the concentration of some trace elements appears decreased (e.g., Se) or increased (e.g., Mo) in both whole blood and serum of HD patients, others show opposite variations in whole blood and serum (e.g., Co and Mn). The whole blood/serum concentration ratio of trace elements studied differed between HD patients and controls. The ratios were higher in HD patients than in controls for Pb (350 vs. only 220), Mn (21 vs. 15) and Zn (12 vs. 8.5). In contrast, ratios were lower in HD patients compared to controls for Co (0.8 vs. 1.7) and Mo (0.8 vs. 1.3).

This study has some limitations. First, it does not include data on the aetiology of CKD, which may significantly affect circulating trace element concentrations [54]. Second, the study did not collect information on the duration of chronic HD, which can also affect circulating trace element concentrations [93]. Third, data on dietary intake of the trace elements analysed were not collected. Studies investigating the effect of dietary Zn supplementation on serum Zn concentration in HD patients have produced conflicting results [94,95]. Furthermore, due to dietary restrictions and uremic anorexia associated with CKD and HD [27,28,29], it is plausible that the dietary intake of trace elements is lower in HD patients. Fourth, CKD is usually accompanied by various comorbidities such as diabetes, hypertension and cardiovascular diseases [2,3] that may influence the concentration of trace elements in blood, but no information was collected on comorbidities of HD patients. Lastly, anaemia is a common condition among HD patients [96,97]. Since most trace elements are not evenly distributed between plasma and blood cells, a lower haematocrit affects their concentration in whole blood. These issues should be addressed in future studies. 

## 5. Conclusions

This study confirms that HD patients tend to present significant trace elements imbalances compared to individuals with normal renal function. By determining the concentration of a wide panel of trace elements in both serum and whole blood, it was shown that chronic HD can affect intra- and extracellular blood compartments differently. Thus, while the Se concentration is decreased in both whole blood and serum of HD patients, Co is increased in serum but decreased in whole blood, and Mn is decreased in serum but increased in whole blood. Future studies should further investigate the mechanisms involved in trace element imbalances found in HD patients, including eventual changes at the subcellular level.

## Figures and Tables

**Table 1:** Mean (SD) and [range] for the trace element concentrations (µg/L) in whole blood of haemodialysis patients and controls. Hedges’ g was used to estimate the effect size of haemodialysis on trace element concentration.

Trace Element	Haemodialysis	Controls	Hedges’ g (95% CI)	*p*-Value
Li	2.66 (1.92) [0.55–8.71]	1.07 (0.81) [0.25–3.43]	1.37 (1.00–1.75)	<0.001
B	133 (62) [45–405]	46 (23) [10–105]	2.31 (1.89–2.71)	<0.001
Mn	8.4 (2.3) [4.1–14.9]	7.6 (1.5) [4.5–11.0]	0.35 (0.02–0.67)	0.025
Co	0.188 (0.062) [0.057–0.366]	0.226 (0.049) [0.146–0.364]	−0.75 (−1.09–−0.40)	<0.001
Ni	1.82 (0.46) [0.91–2.98]	1.53 (0.69) [0.71–3.25]	0.71 (0.38–1.04)	<0.001
Cu	843 (138) [592–1200]	955 (167) [512–1365]	−0.72 (−1.05–−0.39)	<0.001
Zn	5360 (755) [3764–7341]	5767 (2249) [2254–9756]	−0.04 (−0.35–0.28)	0.347
Se	123 (19) [75–169]	138 (20) [92–184]	−0.76 (−1.09–−0.43)	<0.001
Rb	1142 (221) [635–1604]	2457 (670) [1087–3993]	−2.97 (−3.42–−2.51)	<0.001
Sr	29.9 (4.0) [21.6–40.4]	17.6 (6.7) [7.3–35.2]	2.40 (1.99–2.82)	<0.001
Mo	2.48 (1.32) [0.07–5.84]	0.80 (0.26) [0.30–1.50]	1.53 (1.16–1.90)	<0.001
Cd	1.00 (0.32) [0.38–1.86]	0.299 (0.136) [0.094–0.655]	3.31 (2.81–3.81)	<0.001
Pb	100 (24) [42–158]	21.1 (14.2) [5.8–77.8]	4.13 (3.57–4.68)	<0.001

**Table 2:** Mean (SD) and [range] for the trace element concentrations (µg/L) and Cu/Zn ratio in serum of haemodialysis patients and controls. Hedges’ g was used to estimate the effect size of haemodialysis on trace element concentration.

Trace Element	Haemodialysis	Controls	Hedges’ g (95% CI)	*p*-Value
Li	2.89 (1.75) [0.70–7.92]	1.19 (0.76) [0.25–2.94]	1.56 (0.96–2.15)	<0.001
B	89 (42) [22–211]	34 (17) [15–64]	1.88 (1.28–2.47)	<0.001
Mn	0.40 (0.10) [0.22–0.65]	0.52 (0.11) [0.36–0.76]	−1.16 (−1.72–−0.58)	<0.001
Co	0.23 (0.11) [0.12–0.72]	0.130 (0.037) [0.094–0.214]	1.67 (1.09–2.26)	<0.001
Ni	2.80 (0.95) [0.90–5.50]	1.09 (0.25) [0.78–1.58]	1.90 (1.25–2.53)	<0.001
Cu	671 (150) [341–1046]	812 (139) [621–1070]	−0.93 (−1.49–−0.40)	<0.001
Zn	448 (84) [271–658]	681 (114) [475–876]	−2.59 (−3.23–−1.95)	<0.001
Se	62 (13) [32–90]	82 (15) [58–103]	−1.43 (−1.99–−0.87)	<0.001
Rb	87 (24) [34–146]	115 (19) [89–145]	−1.14 (−1.71–−0.57)	<0.001
Sr	29.0 (4.4) [19.7–39.8]	22.3 (7.6) [10.5–36.0]	1.32 (0.76–1.88)	0.003
Mo	3.20 (1.48) [0.16–6.79]	0.61 (0.42) [0.026–1.35]	1.87 (1.27–2.45)	<0.001
Cd	0.038 (0.016) [0.008–0.080]	0.013 (0.005) [0.008–0.018]	2.64 (1.97–3.27)	<0.001
Pb	0.287 (0.149) [0.025–0.755]	0.096 (0.083) [0.021–0.317]	2.55 (1.88–3.20)	<0.001
Cu/Zn	1.54 (0.43) [0.76–2.54]	1.22 (0.25) [0.77–1.65]	0.80 (0.26–1.34)	0.004

## Data Availability

All data related to this study are presented in the article or supplementary material.

## Supplementary Materials

The following supporting information can be downloaded at https://www.mdpi.com/article/10.3390/nu15081912/s1, Table S1: Whole blood and serum analysis—quality control data; Table S2: Limits of detection.
