# Supplementary material for: Further Evidence on Trace Element Imbalances in Haemodialysis Patients—Paired Analysis of Blood and Serum Samples
Source: Nutrients. 2023 Apr 15;15(8):1912. doi: 10.3390/nu15081912 (PMC10145991; doi:10.3390/nu15081912)
Supplement: Supplementary file 1 [file nutrients-15-01912-s001.zip › nutrients-2243537-supplementary.pdf]

Table S1. Whole blood and serum quality control (µg/L).

**Whole Blood Seronorm L1 – lot 1406263**													
	Li	B	Mn	Co	Ni	Cu	Zn	Se	Rb	Sr	Mo	Cd	Pb
**n**	8	8	8	8	8	8	8	8	8	8	8	8	8
**Mean**	0.39	162	19.3	0.24	1.41	600	3610	66	1408	39	0.75	0.28	9.8
**SD**	0.06	16	1.6	0.05	0.28	31	286	5	40	2	0.07	0.03	0.2
**Analytical Value**	0.36	180	18.4	0.20	1.38	640	4300	60	1350	37	0.51	0.28	9.9
**95% CI**	-	-	14.7 – 22.1	0.12 – 0.28	1.10 – 1.66	510 – 760	3400 – 5200	48 – 72	-	-	0.41 – 0.61	0.17 – 0.40	7.9 – 11.9
**Whole Blood Seronorm L2 – lot 1406264**													
	Li	B	Mn	Co	Ni	Cu	Zn	Se	Rb	Sr	Mo	Cd	Pb
**n**	8	8	8	8	8	8	8	8	8	8	8	8	8
**Mean**	0.43	206	32.2	5.13	14.9	1217	7436	162	1412	39	5.09	5.02	298
**SD**	0.06	12	1.9	0.28	0.9	77	1178	13	39	3	0.18	0.38	9
**Analytical Value**	0.33	158	31.4	5.18	15.9	1340	7100	161	1410	36	5.31	5.01	337
**95% CI**	-	-	25.1 – 37.7	4.13 – 6.22	12.7 – 19.1	1070 - 1600	5700 – 8500	128 – 193	-	-	4.24 – 6.37	4.00 – 6.02	269 - 405
**Whole Blood Seronorm L3 - 1509408**													
	Li	B	Mn	Co	Ni	Cu	Zn	Se	Rb	Sr	Mo	Cd	Pb
**n**	8	8	8	8	8	8	8	8	8	8	8	8	8
**Mean**	0.50	392	35.7	9.7	10.5	1820	8277	206	1326	42	7.1	9.1	344
**SD**	0.10	32	2.8	1.0	0.8	224	1470	43	67	4	0.5	0.8	43
**Analytical Value**	0.73	379	33.3	10.3	11.0	2080	8060	198	1180	37	6.2	9.9	362
**95% CI**	-	-	26.6 – 39.9	8.3 – 12.4	8.8 – 13.3	1660 – 2050	6440 – 9680	158 – 238	-	-	4.9 – 7.4	7.9 – 11.9	289 - 434
**Serum Seronorm L1 – lot 1309438**													
	Li	B	Mn	Co	Ni	Cu	Zn	Se	Rb	Sr	Mo	Cd	Pb
**n**	3	3	3	3	3	3	3	3	3	3	3	3	3
**Mean**	4823	58	9.2	1.08	5.71	994	1008	86	4.5	88	0.93	0.13	0.39
**SD**	226	15	0.1	0.06	0.26	36	35	2	0.2	3	0.22	0.03	0.06
**Analytical Value**	5261	-	9.9	1.12	5.64	1066	1057	86	4.4	95	0.76	0.13	0.40
**95% CI**	4202 - 6302	-	7.9 – 11.9	0.67 – 1.57	3.38 – 7.90	852 – 1281	844 – 1269	51 – 120	-	-	-	-	-
**Serum Seronorm L2 – lot 1309416**													
	Li	B	Mn	Co	Ni	Cu	Zn	Se	Rb	Sr	Mo	Cd	Pb
**n**	3	3	3	3	3	3	3	3	3	3	3	3	3
**Mean**	9038	72.5	13.8	2.81	9.2	1722	1451	134	8.6	101	1.24	0.14	0.51
**SD**	73	9.1	0.4	0.15	0.7	95	51	2	0.1	4	0.07	0.04	0.05
**Analytical Value**	9689	82.1	14.5	3.05	9.0	1925	1532	136	8.6	110	1.21	0.14	0.66
**95% CI**	7739 – 11639	-	11.6 – 19.4	2.13 – 3.97	7.9 – 11.9	1538 – 2312	1223 – 1840	95 – 176	-	-	-	-	-

Table S2. Limits of Detection (µg/L).

	Li	B	Mn	Co	Ni	Cu	Zn	Se	Rb	Sr	Mo	Cd	Pb
**Serum**	0.042	1.0	0.061	0.015	0.099	0.13	0.50	3.1	0.049	0.052	0.036	0.011	0.006
**Whole Blood**	0.019	0.63	0.055	0.018	0.23	0.15	0.80	3.2	0.073	0.029	0.039	0.028	0.024
